# 有序多孔材料在色谱分离分析领域的应用

**DOI:** 10.3724/SP.J.1123.2024.05032

**Published:** 2025-06-08

**Authors:** Zhaoxu WANG, Duanda WANG, Shutao WANG, Yongyang SONG

**Affiliations:** 1.中国科学技术大学苏州高等研究院，江苏 苏州 215123; 1. Suzhou Institute for Advanced Research，University of Science and Technology of China，Suzhou 215123，China; 2.中国科学院理化技术研究所，北京 100190; 2. Technical Institute of Physics and Chemistry，Chinese Academy of Sciences，Beijing 100190，China; 3.中国科学院大学，北京 100049; 3. University of Chinese Academy of Sciences，Beijing 100049，China

**Keywords:** 有序多孔材料, 色谱分离分析, 框架材料, 自组装, 聚合物, 综述, ordered porous materials （OPMs）, chromatography separation and analysis, framework materials, self-assembly, polymer, review

## Abstract

色谱作为一种高效率、高选择性的分离技术，拥有广阔的应用范围和发展前景。色谱柱的固定相是色谱技术中最核心的组成部分。研发具备高分辨分离性能的先进固定相材料，一直是该领域的研究热点。有序多孔材料（OPM）凭借其孔径的精确可控性和孔隙结构的规整排列，能够精确筛分不同尺寸和形状的分子，同时减少分子在流动路径中的无序扩散，从而克服了传统色谱材料在分离精度上的限制，有效解决了科研和工业领域在原料及产物纯化方面所面临的难题。过去的几十年间，科研人员已成功开发出多种新型OPM，这些材料被用作色谱柱的固定相基质，实现了对同系物、异构体和同位素等性质相近物质的高效且快速的分离。本文首先从理论层面阐述了有序多孔结构对色谱分离柱效率及分离度的影响，为OPM在色谱固定相中的应用提供了理论支撑。接着，文章综述了包括金属有机框架（MOF）、共价有机框架（COF）、多孔有机笼（POC）、介孔二氧化硅、嵌段共聚物（BCP）组装材料以及高内相乳液聚合物（PolyHIPE）在内的多种类型OPM在色谱分离分析领域的研究进展。最后，文章探讨了当前色谱OPM所面临的挑战，并对未来的发展方向进行了展望。

## Abstract (en)

Chromatography， a highly efficient and selective separation technology， is broadly applicable and exhibits a range of developmental prospects. The stationary phase of a chromatography column is the most important component of chromatography； hence， the development of advanced stationary-phase materials that exhibit highly resolved separation performance is a continuing research hotspot in this field. In this regard， ordered porous materials （OPMs） are advantageous owing to their precisely controllable pore sizes， morphologies， and regularly arranged pore structures， which are capable of accurately sieving molecules of different sizes and shapes， and reducing disordered molecular diffusion in the flow path. Such materials overcome the limitation of separation accuracy of traditional chromatographic materials， and effectively solve the problems faced by scientific research and industry in the purification of raw materials and products. Over the past few decades， a variety of new OPMs have been developed and used as stationary-phase matrices in chromatography columns. These materials have efficiently and rapidly separated homologues， isomers， isotopes， and other substances with similar properties， and have delivered excellent chromatographic separation and analysis results. In this review， we first discuss the influence of ordered porous structures on column efficiency and resolution during chromatographic separation from a theoretical perspective， which provides a basis for the use of OPMs as stationary phases in chromatography. This review then summarizes research progress on several different OPM types for use in chromatographic separation and analysis applications， including metal organic frameworks （MOFs）， covalent organic frameworks （COFs）， porous organic cages （POCs）， mesoporous silica materials， block copolymer （BCP） assemblies， and high internal-phase emulsion polymers （PolyHIPEs）. The review concludes by discussing current challenges faced by chromatographic OPMs as well as directions for future development.

色谱是一种用于分离气体或液体混合物的技术，与传统的热分离方法（如精馏等）相比，色谱展现出高效率和高选择性的优势，因此得到了广泛应用^［1，2］^。至今，已有多种色谱方法被开发出来，包括高效液相色谱法（HPLC）、气相色谱法（GC）和超临界流体色谱法（SFC）等^［2］^，这些方法成功实现了同系物、异构体、手性化合物，以及诸如核苷酸、多糖、蛋白质等生物大分子的有效分离。一个典型的色谱设备通常由进样系统、色谱柱（含固定相）以及检测系统等多个部分组成。其中，色谱柱内的固定相是保持静止并对样品产生保留作用的关键部分，作为设备的核心组件，它对混合物的分离分析效果起着至关重要的作用。传统的色谱柱固定相主要由硅胶颗粒构成，因其易于修饰且操作简便、快捷，而获得了广泛的商业化应用。然而，由于硅基材料的耐酸碱性较差，聚苯乙烯、琼脂糖等聚合物材料也常被用作色谱固定相，从而拓宽了色谱分离分析的应用领域。尽管如此，这些聚合物材料也存在机械强度较低、易溶胀变形等问题。近年来，随着差异性应用场景的不断增多，对固定相材料的需求也日益提高^［3‒6］^。因此，探寻适用于色谱分离分析的新型先进固定相材料，已成为当前相关领域的研究热点。另一方面，固定相材料的结构对色谱性能有着显著的影响。与无孔颗粒相比，多孔材料因其较高的比表面积和丰富的表面相互作用位点而占据优势。多孔结构不仅能有效降低渗透压，还能缩短扩散路径，进而减少扩散率，提升色谱柱的效率^［7］^。

有序多孔材料（ordered porous material， OPM）凭借其均匀的孔径、规整的孔隙排列、可调的骨架结构以及高比表面积，在吸附分离领域展现出了卓越的性能^［8‒10］^。近20年来，一系列新型OPM（如金属有机框架（MOF）、共价有机框架（COF）、多孔有机笼（POC）、介孔二氧化硅、嵌段共聚物（BCP）组装材料和高内相乳液（high internal phase emulsion，HIPE）聚合物（PolyHIPE）等）已被用作固定相基质，并成功用于色谱分离分析^［2，11］^。与传统多孔材料相比，OPM能够实现更高精度的尺寸控制，这对于分离化学性质极为相似的分子（如同系物^［12］^和异构体^［13‒15］^）具有显著优势，进而提升了色谱分析的分辨率和选择性。

尽管新型色谱材料蓬勃发展，但在理论模型与应用进展方面，关于OPM作为色谱固定相的系统性论述与总结尚显不足。本文从理论层面阐述了OPM对色谱分离效率及分离度的影响，随后介绍了几种常见OPM在色谱分离分析领域的研究进展，并对其未来的发展趋势进行了展望。

## 1 理论依据

在多孔材料中，混合物的传输主要依赖于以下3种不同的分离机制：（1）基于不同吸附质与吸附剂间相互作用产生的热力学平衡来实现分离；（2）基于各组分扩散速率的不同而导致的动力学分离；（3）通过尺寸和/或形状排除效应来进行分子筛分^［16］^。在色谱分析中，不同物质的分离主要取决于它们在流动相与固定相之间的分配差异。OPM凭借其均匀的孔径尺寸和规则的孔道路径，不仅便于筛分分子，还有助于降低分子在色谱分离过程中的无规则扩散，从而提升流动相的传质效率。然而，孔隙性质对不同固定相形态的色谱柱分离效果有所差别。下文将分别阐述多孔结构的有序度对填充柱和整体柱（图1a）两种典型色谱柱的分离性能影响。

**图1:**
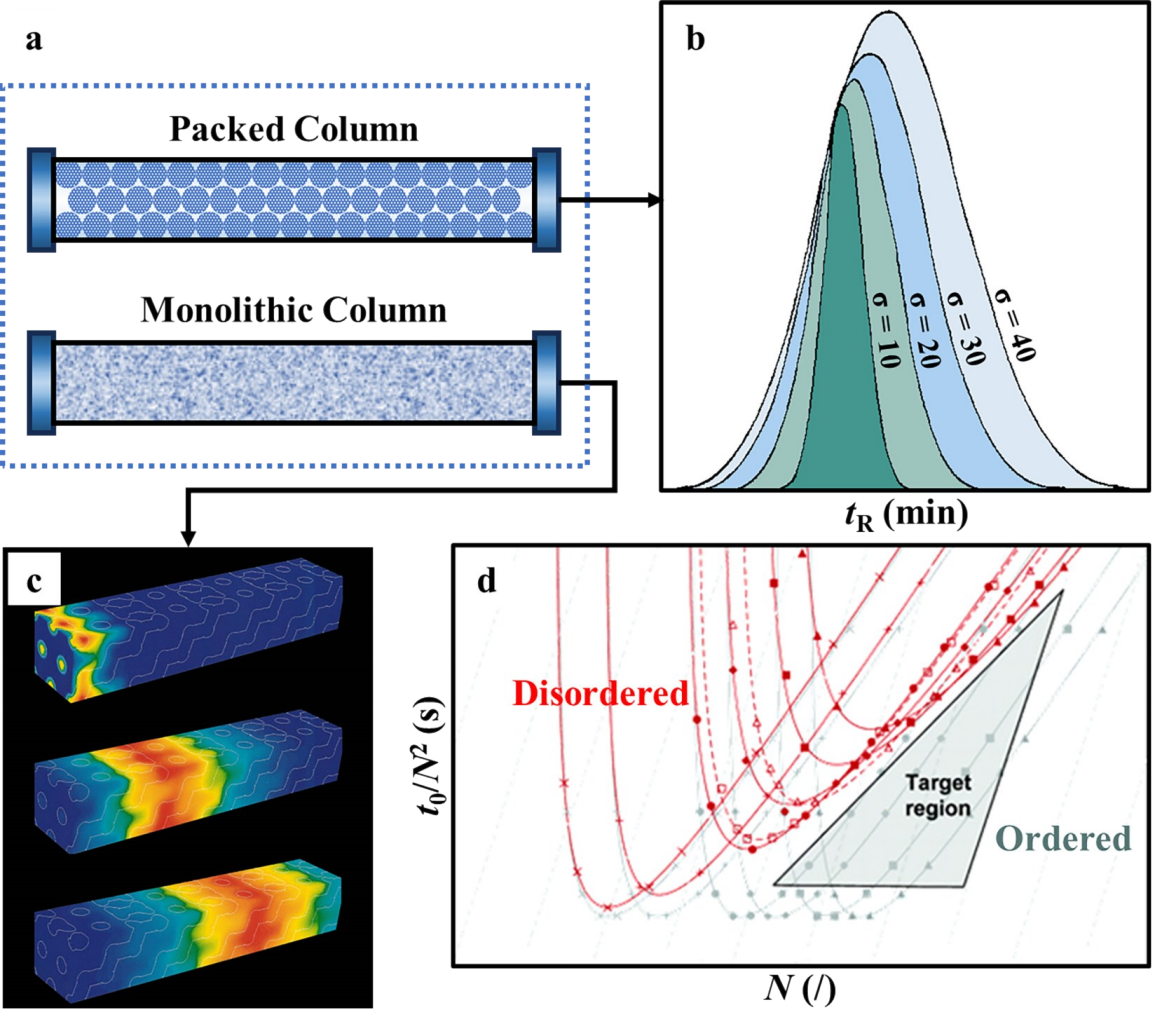
（a）多孔填充柱与整体柱示意图；（b）不同孔径分布宽度所对应的保留时间分布概率^［17］^；（c）TSC中示踪剂的模拟传输过程^［18］^；（d）有序（灰色曲线）和无序（红色曲线）结构整体柱的动力学曲线^［19］^

### 1.1 有序度对填充柱的影响

填充柱的固定相由颗粒构成，装填过程快速且简便。然而，这些颗粒在色谱柱中通常是随机堆积的，颗粒之间无规空隙的存在使得对填充柱内传质过程的分析较为复杂。颗粒填充柱的柱效可通过如下公式进行计算^［20］^：


$$H=\frac{1}{\frac{1}{C_{e}d_{p}}+\frac{D_{m}}{C_{m}d_{p}^{2}u}}+\frac{C_{d}D_{m}}{u}+\frac{C_{sm}d_{p}^{2}u}{D_{m}}$$ (1)

其中，*H*为塔板高度（m），用以表征色谱柱的效率；*d*_p_为填料粒径（m）；*u*为流动相线速度（m/s）；*D*_m_为溶质扩散系数（m^2^/s）；*C*_e_、*C*_m_、*C*_d_和*C*_sm_分别代表涡流扩散、流动相传质、纵向扩散以及颗粒内传质对柱效的贡献。一般认为，多孔颗粒的孔径均匀程度会影响*C*_sm_等系数的数值，但其对填充柱的柱效贡献小于流速、孔隙率、粒径尺寸及其分布等关键因素^［21］^，通常可以忽略不计。然而，在尺寸排阻色谱法（SEC）中，孔径的尺寸分布却扮演着至关重要的角色。1968年，Carmichael^［17］^通过理论分析与实验验证，揭示了凝胶渗透色谱中孔径尺寸分布与聚合物分离效果之间的关联。对于任意孔径分布，单分散线性聚合物在色谱柱中保留时间的一般表达式如下：


$$t_{R}-t_{0}=Ct_{0}\sum_{i=1}^{n}a_{i}\frac{\lambda_{1i}}{\lambda_{2i}}$$ (2)

$$\sum_{i=1}^{n}a_{i}=1$$ (3)

其中，*t*_R_为聚合物的保留时间（s）；*t*_0_为聚合物分子质量足够高时所对应的最短保留时间（s）；*C*为单位色谱柱长度的捕获位点浓度；*λ*_1_ *_i_* 为聚合物分子被捕获到第*i*个孔径时的速率常数，*λ*_2_ *_i_* 为聚合物分子从第*i*个捕获部位（孔径）洗脱进入流动相时的速率常数，*λ*_1_ *_i_* 与*λ*_2_ *_i_* 的比值称为捕获概率；*a_i_* 代表尺寸为*r_i_* 的孔径分布概率，若假设其服从高斯分布，则存在如下关系式：


$$a_{i}=\frac{1}{\sqrt[{}]{2\pi \sigma^{2}}}exp\;\left[\frac{-{\left(r_{i}-r_{av}\right)}^{2}}{2\sigma^{2}}\right]$$ (4)

其中，*r_i_* 为第*i*个孔径的尺寸（m）；*r*_av_为平均孔径（m）；*σ* ^2^为孔径分布方差。图1b展示了在填充柱中，不同孔径分布宽度所对应的保留时间分布概率情况；Carmichael^［17］^证明，宽的孔径分布会显著影响分子的保留行为，导致保留时间延长并使得时间分布变宽。此外，Bacskay等^［22］^通过构建特征函数的方法，推导出了尺寸排阻效应的平衡分配系数（*K*_SEC_），并得出了相似结论，进一步验证了孔径分布对颗粒间传质过程的影响。综上所述，有序度较高的多孔色谱填料具有更均一的孔径分布，能够有效减少分子在填充柱中的无规则扩散，从而提升色谱分离的效率。

### 1.2 有序度对整体柱的影响

整体柱通常是通过在色谱柱内部原位聚合反应性单体等方法制备而成，具有高度连通的一体式网络结构。与填充柱相比，整体柱展现出更高的孔隙率和更强的渗透性，流动相在柱内受到的阻力较小，流速相应加快，从而有利于实现快速的传质过程^［7］^。在整体柱中，孔隙取代了颗粒成为固定相的基本构成单元，因此孔径的尺寸及其分布等特性将直接影响整体柱网络的均匀程度，并对柱效和传质过程产生更为明显的作用。Leinweber等^［23］^提出了一种方法，用于比较整体柱与颗粒填充柱的色谱性能。该方法通过将整体柱中的孔径转化为等效的颗粒（球体）尺寸，以此作为衡量色谱径向渗透性和轴向色散率的特征长度。通过复杂的函数表征，Leinweber等认为，尺寸足够大且呈单峰分布的孔隙能够提供更有利于分子流通的通道，从而减少分子的轴向扩散，提升色谱分离效率。因此，具有单峰孔径分布的聚合物整体柱常被应用于下游的精细分离领域，例如蛋白质和DNA等生物大分子的纯化过程。相比之下，二氧化硅整体材料由于在溶胶-凝胶工艺中的成核和生长机制，会形成独特的双峰孔径分布结构，更适用于中、低分子质量物质的分离^［7，23］^。

Vervoort等^［18，24］^针对非均质整体柱，构建了一种四面体骨架柱（TSC）的结构模型，并进行了理论模拟。他们通过对比完美有序的理想状况与实际状况的差异，阐述了孔径均匀性对色谱性能的影响。如图1c所示，Vervoort等^［18］^利用流体动力学计算软件（CFD）模拟了TSC中示踪剂的传输过程，并详细计算了谱带展宽的相关数据。其中，用以表征折合塔板高度的公式如下：


$$h=Av^{1/3}+\frac{B}{v}+C$$ (5)

式中*h*为单位长度的折合塔板高度，*v*为折合流速，A、B、C则为常数。式中A项代表了流经孔隙的非均质性对谱带展宽的贡献。拟合结果显示，在完美有序的整体柱中，A项的数值相较于无序的实际二氧化硅整体柱大约低一个数量级，这有力证明了OPM整体柱在降低折合塔板高度和分离阻抗等方面具有显著优势。此外，Billen等^［19，25］^从孔隙位置分布的角度对整体柱的有序度进行了模拟研究，并进一步证实，随着无序程度的增加，色谱柱的效率会相应降低。具有完美有序结构和无序结构的整体柱动力学性能曲线如图1d所示，其中*N*为表征色谱柱效率的塔板数（块/m），*t*_0_为流动相流经色谱柱所消耗的死时间（s）；灰色曲线代表具有完美有序结构的整体柱，红色曲线代表实际二氧化硅整体柱。由图1d可知，灰色曲线的最低值位置不随柱效提升而改变，显示出完美有序结构的稳定动力学性能；红色曲线在图像右侧呈现出三角形禁带，证明无规阵列色谱的动力学性能存在极限，只能通过提高有序度来改善。在相同柱效（*N*=5×10^4^ 块/m）条件下，无序色谱柱的分析时间与有序色谱柱相比，存在数量级上的差异^［19］^，证明了采用OPM作为整体柱固定相在动力学方面的显著优势。

## 2 应用于色谱固定相的OPM

一般而言，多孔材料可以根据孔径尺寸大小分为微孔（<2 nm）、介孔（或中孔）（2~50 nm）以及大孔（>50 nm）材料。不同材料所对应的孔径尺寸范围往往存在差异，如MOF、COF、POC等材料，其孔隙是通过化学键构筑的，因此具有分子尺度的微孔；多孔二氧化硅材料通常由纳米尺寸的模板煅烧制备得到，从而呈现出介孔结构；通过自组装或液体模板固化形成的聚合物材料则具有从介孔至大孔更宽范围的孔隙结构。在分离应用中，不同孔径的材料为各种尺度的物质分离提供了良好的选择性，尤其是在色谱分析领域，多种孔道尺寸控制精确的OPM已被广泛用作色谱固定相材料。

### 2.1 MOF材料

MOF材料是一类由无机物中心（金属离子或团簇）与有机物配体通过自组装配位形成的有机-无机杂化材料，其周期性网络结构的晶体内部存在大量分子尺度的均匀规整孔隙^［26］^。这种材料通过强化学键连接，因此具有较高的热稳定性、化学稳定性和永久孔隙率。自20世纪90年代以来，金属离子与有机配体结合形成的配位分子已得到大量报道，并被证明具有稳定的多孔结构^［27，28］^。迄今为止，新合成开发的MOF材料种类已超过20 000种。这些MOF材料凭借超高的孔隙率（自由体积可达90%）、极大的比表面积（超过7 000 m²/g）以及优异的主客体特性等特点^［2，29］^，在气体存储和分离等领域得到了广泛应用^［30，31］^。

凭借精确的孔径和形状控制能力，MOF材料在分离那些性质稳定且组成相近的分子时展现出独特的优势，特别是在同系物和异构体的分离中表现出了卓越性能^［14，32］^。例如，李静课题组^［12，13，33‒36］^验证了MOF材料对多种直链、单支链及多支链烃类混合物的色谱分离有效性。MOF材料具有分子级别的微孔结构，可精确匹配吸附物的形状和动力学直径，从而在异构体之间实现显著的吸附选择性差异，这种差异跨越了不同的数量级。这一特性为石化行业中原料及产品的纯化提供了理想的解决方案。进一步地，在对映异构体的分离过程中，由于局部手性相较于拓扑手性起着更为关键的作用，MOF材料所采用的较为温和的制备方法能够有效避免去除模板或活化过程对结构的潜在破坏。因此，MOF材料在分离对映异构体方面通常能够展现出比沸石等传统无机材料更好的分离效果^［30，37，38］^。此外，MOF材料还具备高度可调控的骨架结构，这一特性使其相较于许多刚性分离材料展现出额外的优势。柔性MOF的开发使得材料能够对外部刺激（如吸附物、压力和温度等）作出响应，通过精确调控孔径和形状来适配目标客体分子，从而优化了特定气体混合物的分离性能^［15，34，39］^。Yu等^［15］^利用一种具有柔性骨架的钙基MOF材料（HIAM-203），成功实现了对二甲苯及其异构体之间的高选择性分离。该MOF材料对二甲苯异构体的吸附行为展现出温度依赖性，特别是在工业操作温度（120 ℃）下，通过尺寸排阻效应，能够在异构体混合物中高效地分离出对二甲苯。经计算，其对邻二甲苯和间二甲苯的分离选择性分别为378.8和860.5，充分展示了该材料在异构体分离方面的卓越能力。

除小分子之外，近年来MOF材料也被应用于分离高分子聚合物。Mizutani等^［40］^首次报道了一种利用MOF填充柱对末端功能化聚乙二醇（PEG）进行色谱分离的方法。该方法通过MOF材料实现了对不同末端官能团的精确识别，从而有效区分各种聚合物。末端基团较大的PEG由于无法插入到MOF的纳米孔中，在色谱柱中没有保留，注入后会被迅速洗脱，其洗脱时间略短于溶剂的洗脱时间。相反，具有羟基、甲氧基和甲基丙烯酸酯等较小末端基团的PEG分子能够进入MOF的通道，并根据其化学性质的差异展现出独特的保留时间，呈现出可清晰区分的洗脱峰。这种MOF材料具有出色的高分子识别能力，作为色谱固定相，可成为分离各种遥爪聚合物的有力工具。然而，当前MOF材料仍面临吸附容量与选择性难以兼顾，以及热稳定性和化学稳定性不足等问题。为了拓展其在分离领域的应用，与其他材料复合已成为一种常用的解决策略^［41，42］^。

### 2.2 COF材料

COF材料是一类由有机分子通过共价键连接而成的结晶多孔聚合物材料，具有高规整度的有序孔隙排布^［43‒45］^。2005年，Côté等^［46］^基于框架化学的理念，首次成功合成了COF-1和COF-5这两种代表性的COF材料，并揭示了它们独特的多孔晶体结构。自此以后，众多COF结构不断涌现并被广泛报道，包括硼酸类、亚胺类、三嗪类以及吩嗪类等多种共价键类型的COF材料^［47］^。与其他无机或杂化多孔材料相比，COF材料完全由H、B、C、N、O等轻质元素构成，具有更低的密度。同时，COF材料中不含重金属离子，因此展现出更高的生物相容性和更低的毒性，从而在生物医学领域的潜在应用中获得了无可替代的优势^［47，48］^。

COF因具备高比表面积、永久孔隙率、明确的晶体结构以及对孔隙环境的精确调控能力，在分离领域展现出了巨大的应用潜力^［16，49］^。与MOF相比，COF材料的孔径较大，且在构建0.6 nm以下的超微孔方面面临挑战，因此不太适用于气体等小分子物质的分离。然而，COF材料独特的组成赋予了其出色的可修饰性和可设计性。其拓扑结构所产生的*π-π*堆积、氢键等相互作用以及丰富的活性位点，共同促使COF展现出卓越的分子吸附性能，从而在一定程度上克服了尺寸排除效应的限制。在色谱分离领域，COF材料已被广泛应用于CO_2_的捕获^［50］^、同位素富集^［51］^以及小分子有机物的分离^［52，53］^等多个方面。Li等^［53］^制备了一种亚胺基TAPB-BTCA COF，并首次将其作为高效毛细管电色谱（CEC）的多孔固定相。这种新型色谱柱对烷基苯、氯苯、香兰素、对羟基苯甲酸酯、苯酚和相关酚类化合物等6种小分子化合物以及非甾体抗炎药物表现出了优异的分离性能，色谱峰形良好且柱效高（最高可达293 363 块/m），能够实现基线分离。在手性对映体的拆分过程中，手性COF材料为色谱分离提供了至关重要的手性微环境和丰富的相互作用位点^［37，54‒56］^。Chen等^［56］^合成了一种由手性分子甲基化环糊精（MCD）和谷胱甘肽（GSH）共同修饰的COF材料（GSH-MCD COF），并将其用作HPLC的固定相。其中，MCD对手性醇和胺具有良好的识别能力，而GSH对氨基酸和有机酸等化合物具有较强的选择性，二者的结合提供了一个受限的物理化学微环境，能够产生多手性协同效应，从而扩展了可分离的手性对映体范围。此外，Chen等^［56］^还测试了该COF固定相对多种手性芳香醇、芳香酸、酰胺、氨基酸和有机酸的分离效果，结果显示，在色谱图中这些化合物均展现出了清晰的分离峰，并且获得的分离因子（*α*）和分离度（*R*_s_）相较于商用色谱柱均有所提升。这一发现有力验证了协同多手性COF在分离和识别性能上的优越性。Zhang等^［57］^将一系列生物分子（包括氨基酸、肽和酶等）固定到非手性COF材料上，实现了对多种外消旋体（如DL-苏氨酸、DL-色氨酸、氧氟沙星及美托洛尔等）的特异性分离，从而为生物医药分离领域开辟了一条可行途径。经HPLC检测，各种外消旋体样品在色谱图中均呈现出两个清晰、无重叠且无拖尾现象的狭窄信号峰，同时展现出良好的*α*和*R*_s_，这充分证明了生物分子的高级结构在手性识别和分离过程中的重要性。目前，更高的机械强度和稳定性仍是COF固定相合成过程中所追求的目标之一，可通过交联^［58］^或与金属离子^［59］^、二氧化硅、Fe_3_O_4_等无机纳米粒子^［60］^复合来提高COF材料的性能。

### 2.3 POC材料

POC是一类具有笼状结构的多环化合物，其内部包含一个允许客体分子进入的空腔，该空腔通过互穿孔隙与外部环境相连^［61，62］^。2009年，Tozawa等^［61］^首次报道了POC，他们合成了一系列具有高永久孔隙率的四面体［4+6］ C=N键有机笼。与MOF和COF等长程有序且具有高结晶度的材料相比，POC主要依据动态共价化学（DCC）原理进行组装，在溶解性方面具有独特优势，能够在不牺牲可加工性的前提下表现出所需的选择性。通过分子间作用力的结合，离散的POC分子可以像模块化有机构件一样堆积在一起，形成孔隙网络，这为构建结构多样化的功能材料提供了一种新颖的途径^［63］^。

由于具有高比表面积、永久孔隙率、可调结构以及良好的热稳定性，POC材料被广泛应用于相似尺寸的气体^［64］^、同位素^［65］^以及同分异构体^［66，67］^的精细分离中。在色谱领域，POC材料主要用作GC和CEC的固定相涂层材料^［68］^。Su等^［64］^将新型POC材料（CPOC-301）作为吸附剂，成功地从乙烷混合物中高效分离出高纯度乙烯（*α*为1.3），为石化行业中碳氢化合物的高选择性纯化开辟了一条新路径。Li等^［66］^合成了一种羟基功能化的手性POC-1材料，并将其作为GC固定相。实验测得该固定相的*N*高达3 300 块/m。利用该色谱柱，成功实现了包括醇、酯、酮、醚、卤代烃和有机酸等在内的39种外消旋体的高分辨率分离，其中多种消旋体达到了基线分离效果，峰形尖锐。这一成果进一步拓宽了POC基色谱柱在分离分析方面的应用范围。

近十几年来，对有机笼内空腔结构的设计与优化工作从未间断。增大空腔体积被视为拓宽POC材料可分离目标分子范围的有效途径，预示着其在更大尺寸分子负载等领域具有潜在的应用前景^［69］^。面对POC材料结构不稳定、易塌陷等问题，金属有机笼（MOC）^［70］^等多种可替代的笼状材料被不断开发出来。此外，基于有机笼构建笼型COF材料也成为近年来极具开发潜力的策略之一^［71］^。

### 2.4 介孔二氧化硅材料

按照国际纯粹与应用化学联合会（IUPAC）的定义^［72］^，孔径大小为2~50 nm的材料被称为介孔或中孔材料。与MOF和COF等具有分子级别孔径的材料相比，介孔材料因其能够允许蛋白质等大分子通过，有效地填补了亚细胞尺度分离材料的空白，从而在生物医学等领域拓展了应用范围^［73］^。其中，二氧化硅凭借其高比表面积、易于功能化以及较好的生物相容性和化学稳定性等特点，成为生物分子分离等应用中的理想材料^［74］^，并广泛被用作HPLC等色谱技术的固定相。

直径<10 μm的完全纳米多孔二氧化硅微粒是一种优异的纳米多孔色谱材料^［75，76］^。提升色谱分离效果和分辨率的关键在于，要严格控制二氧化硅微球的形态与尺寸稳定性，同时这也对孔径分布提出了更为严苛的要求。1992年，Kresge等^［77］^首次采用液晶作为硬模板，成功制备出了具有有序介孔结构的二氧化硅分子筛。自此之后，利用模板法合成有序介孔二氧化硅微球（mesoporous silica microsphere，MPSM）的研究报道层出不穷^［78］^。Mayer课题组^［79，80］^提出了一种以聚合物多孔微球为模板的新方法，成功合成了孔径分布狭窄且可调的MPSM。利用功能化聚合物作为硬模板，有利于调控MPSM的孔隙参数和引入官能团。现有研究表明，采用具有均匀孔道的介孔二氧化硅填料，在HPLC中成功实现了4种典型蛋白质和11种氨基酸的基线分离（*R*_s_≥2.0），彰显了其卓越的分辨率性能^［79，80］^。近年来，MPSM的表面修饰技术已经发展成为一项成熟的工艺，广泛应用于制造市售分离材料，这一技术有效解决了均质二氧化硅材料在高度特异性分离方面的局限性。目前，多种特殊的官能团或配体分子，如阴/阳离子、亲/疏水基团、聚合物、金属离子、多肽以及蛋白质等，已被成功引入到MPSM中，这些修饰使得MPSM能够适用于不同亲疏水性分子、带电荷分子以及特异性生物分子的吸附与分离，极大地拓展了其应用范围^［11］^。值得一提的是，通过采用更大尺寸的聚合物微球作为模板，可以制备出有序的大孔二氧化硅整体材料^［81］^。该合成工艺涉及自组装方法，具体细节将在后续章节中详细阐述。

### 2.5 BCP组装材料

自组装是制造纳米有序多孔结构材料的一种关键策略^［82‒84］^，它依赖于熵驱动自发地形成稳定的有序结构，这一过程不仅经济、高效，而且操作便捷。BCP可视为一种软模版，能够通过自组装的方式构建出无缺陷、均匀且规整的纳米结构，且模板的去除过程简便甚至无需去除。BCP组装材料在孔径控制方面表现出色，孔径尺寸分布狭窄。通过调整BCP合成及组装过程中的组成、配比等参数，可以灵活地调控其结构和性能。此外，通过提高聚合物分子质量或采用刷形聚合物，还能够获得孔径更大的大孔材料，这是大多数无机物和有机小分子难以实现的特性^［85，86］^。Peinemann等^［87］^提出了一种制备有序纳米尺寸孔薄膜的有效策略，通过将两亲性BCP聚苯乙烯-*b*-聚（4-乙烯基吡啶） （PS-*b*-P4VP）的自组装与非溶剂诱导相分离（NIPS）技术结合，在溶剂与非溶剂的交换过程中，通过选择性溶胀来诱导孔隙的生成。通过自组装得到的薄膜材料对外界环境刺激（如pH变化）具有良好的响应性，能够实现对尺寸相近蛋白质的有效分离。随后，该团队采用相同方法成功制备了有序多孔颗粒，为生物分子和药物的吸附、分离及递送提供了一种理想的选择^［88］^。

自1988年被开发以来^［89］^，基于BCP组装的纳米多孔材料因其孔径均匀可控、结构排列规整等优点，引发了广泛的研究兴趣，并在过滤分离等领域得到了广泛应用^［90，91］^。然而，现阶段BCP组装材料仅在膜分离领域获得了相对成熟的应用^［92，93］^，受限于其较低的机械强度和抗溶剂性能，BCP在色谱分离领域的应用报道相对较少。当前，自组装技术更多地作为一种制备手段，应用于其他类型材料固定相的加工成型工艺中。例如，Li等^［94］^利用典型的聚氧乙烯/聚氧丙烯醚三嵌段共聚物Pluronic F127 （PEO-*b*-PPO-*b*-PEO）的自组装特性，辅助甲基丙烯酸缩水甘油酯（GMA）与乙二醇二甲基丙烯酸酯（EDMA）进行乳液聚合，制备了一种新型的聚合物基HPLC整体材料。该课题组采用冷冻凝胶乳液技术^［95］^，以自组装产生的冰晶为模板，实现了多孔结构的构筑。在此过程中，Pluronic F127既充当了乳液体系的稳定剂又充当了模板控制剂。将该整体材料用作HPLC固定相，能够产生多个清晰的独立样品峰，并成功分离了多种探针分子，且不同批次间的偏差小于2.9%，展现出了良好的重复性，其分离性能优于传统色谱柱。Peng等^［96］^通过两亲性二嵌段糖聚合物（ADG）与Pluronic F127在乳液界面上的协同自组装策略，为双连续形态聚苯乙烯整体材料的合成创造了稳定的界面条件。ADG的引入克服了第二代聚苯乙烯整体材料在实际应用中的局限性，使其表现出优异的生物相容性。将该材料应用于亲水相互作用色谱（HILIC）中，在高速模式下直接分离糖蛋白，结果显示，该材料对牛血清白蛋白（BSA）的非特异性吸附降低了91.58%，同时展现出尖锐、清晰的样品峰和较大的峰间距，这预示着它在分析检测领域具有巨大的应用潜力。

### 2.6 PolyHIPE材料

通过聚合反应获得的部分交联聚合物能够形成高度互连的三维网状多孔结构，其可作为多孔材料应用于色谱固定相中。与大多数二氧化硅整体材料所展现的孔径尺寸双峰分布不同，聚合物基整体材料能够实现孔径尺寸的单峰分布以及孔隙排列的高度有序性。目前，液体模板法^［97‒100］^已成为制备大孔整体聚合物材料的一种常用且有效的方法，它通常利用HIPE作为模板，在乳液体系内完成聚合过程。该方法可将孔径控制在100 nm~2 mm范围内^［101］^，从而制备出具有明确孔隙率和高渗透性的高度多孔材料，也因此激发了科研人员广泛的研究兴趣。

HIPE是一种高黏度的膏状乳液，其内相（即分散相）的体积占比通常超过乳液总体积的74%。当该乳液的外相（连续相）经过聚合并固化后，即可得到具有高度相互贯通多孔结构的PolyHIPE材料。从组成上来看，HIPE多为水包油（O/W）乳液。但在近十几年间，许多新型HIPE体系也被开发出来，如CO_2_/水乳液（C/W）^［102］^以及油包油（O/O）^［103］^、油包离子液体（IL/O）^［104］^等非水乳液。为确保PolyHIPE的孔隙尺寸及结构得到精确控制，在合成过程中，乳液的稳定性显得尤为关键。与传统的表面活性剂相比，高分子质量的两亲性BCP^［105，106］^以及无机纳米粒子^［107，108］^已被证实具有更好的乳液稳定和分散性能。此外，为了克服传统乳液聚合过程中因不可控性而导致的结构均匀性受限问题，多种可控的新型乳液聚合方法逐渐被开发出来^［96］^。

PolyHIPE材料具有孔隙率可控、渗透率高、机械强度良好、稳定性强以及化学性质可定制等显著优点，在分离科学领域受到了日益增长的关注并得到了广泛应用^［109‒113］^。Tunç等^［109］^通过对含有丙烯酸异癸酯（IDA）和二乙烯基苯（DVB）单体的HIPE连续相进行原位聚合，制备了一种新型CEC整体柱。该色谱柱的固定相展现出明确的开孔结构和单分散的微米级孔径分布特点，同时聚合过程中产生的可电离硫酸根基团能够生成电渗流（EOF），从而实现了对不同极性烷基苯的高效分离；其中，*H*最低为5 μm，*N*高达200 000 块/m。Desire等^［112］^在制备整体柱的过程中，采用了高剪切应力乳化技术，有效改善了PolyHIPE材料的径向异质性，并提供了更加均匀的孔径尺寸分布。在反相色谱模式下，Desire等^［112］^研究了核糖核酸酶A、溶菌酶和*α*-糜蛋白酶原A这3种蛋白质标准混合物的分离效果。与采用低剪切速率（300 r/min）色谱柱导致的共洗脱现象相比，高剪切速率（14 000 r/min）色谱柱能够清晰区分这3种蛋白质，并获得了更高的信号峰强度和更窄的峰宽度，证明了色谱分辨率的提升。迄今为止，PolyHIPE材料已在多种色谱分析中得到了广泛应用，包括CEC^［109］^、纳米液相色谱（nano-LC）^［110］^和HPLC^［111］^等，成功实现了有机小分子、CO_2_气体、金属离子以及蛋白质等多种物质的检测与分离^［100］^。随着微流控等前沿技术的不断进步，PolyHIPE材料的制备方法得到了进一步的优化^［114‒117］^。微流控技术可以生成尺寸精度极高、多分散指数极低（PDI<5%）的乳液或泡沫，有利于实现孔径及结构的精准控制。然而，微流控合成方法存在耗时较长和产量相对较低等问题，因此在色谱材料领域的应用尚未被深入探索。

综上所述，包括MOF、COF、POC、MPSM、BCP组装材料和PolyHIPE等在内的多种OPM已被开发用于色谱分离分析领域。这些OPM的孔径范围非常广泛，从亚纳米级的微孔到微米级的大孔均有涵盖，这些典型OPM的几种常见色谱分离应用如表1所示。一般而言，除孔径有序程度外，表面化学性质、颗粒几何形貌及尺寸分布等也是固定相材料的关键参数，是影响色谱性能的重要特征。在上述OPM固定相的合成及加工过程中，模板法等制备技术能够实现材料形貌与结构的精准控制。同时，通过化学合成和自组装方法，可以有效调节材料的表面化学性质。特别是对于整体柱和涂层柱这类非颗粒填充的固定相，它们无需考虑颗粒间的异质性。因此，灵活整合多种材料的优势，合理调控孔隙大小、颗粒尺寸以及表面性质等参数之间的制约与平衡关系，将有望进一步提升色谱分离分析的性能。

**表1:** 用于色谱分离的典型OPM

Materials	Pore type	Pore size distribution	Chemical compositions	Target molecules	Refs.
MOFs	micropore and mesopore	narrow	HIAM-410	hexane isomers	［^14^］
			HIAM-203	xylene isomers	［^15^］
			Zr-abtc， Al-bttotb， Co-fa	naphtha	［^33^］
			Mn（ina）_2_	Xe/Kr	［^39^］
			Zn-ADC， Zn-BDC， Zn-NDC	PEG	［^40^，^42^］
			UiO-66/NH-methacrylate	proteins	［^41^］
COFs	micropore and mesopore	narrow	SP-CA-COF-IM， SP-CA-COF-AM	CO_2_	［^50^］
			pyrdine@COF-1	H_2_/D_2_	［^51^］
			NKCOF-36， NKCOF-37	C_3_H_4_/C_3_H_6_	［^52^］
			TAPB-BTCA COF	alkylbenzenes， chlorobenzenes， phenols， parabens， vanillin， phenolic compounds， non-steroidal anti-inflammatory drugs	［^53^］
			DA-TD COF	terbutaline enantiomer	［^54^］
			GSH-MCD COF	chiral aromatic alcohols， aromatic acids， amides， amino acids， organic acids	［^56^］
			lysozyme⊂COF-1	biomolecules	［^57^］
POCs	micropore and mesopore	narrow	CPOC-301	C_2_H_4_/C_2_H_6_	［^64^］
			CC3 POC	H_2_/D_2_	［^65^］
			POC-1	racemic alcohols， diols， halohydrocarbons， epoxides， esters， lactones， ketones， ethers， organic acids	［^66^］
MPSMs	mesopore	broader	silica	amino acids	［^79^］
			silica	proteins	［^80^］
BCP assemblies	mesopore and macropore	broader	Pluronic F127， Poly（GMA-co-EDMA）	polycyclic aromatic hydrocarbons， basic aromatic amines	［^94^］
			Pluronic F127， ADG	glycoprotein	［^96^］
PolyHIPEs	macropore	broader	Poly（IDA-co-DVB）	alkylbenzenes	［^109^］
			Poly（Sty-co-DVB）	alkylbenzenes	［^111^］
			Poly（Sty-co-DVB）	proteins	［^112^］

PEG： polyethylene glycol.

## 3 总结与展望

得益于精确的孔径尺寸和规整的孔隙排列，OPM作为固定相展现出了优异的分子筛分效应和均匀的扩散路径长度，这预示着它在色谱分离中将有望实现更高的分离度和更出色的选择性。经过数十年的发展，目前以MOF、COF、POC、介孔二氧化硅、BCP组装材料和PolyHIPE等为代表的OPM固定相，在同系物、异构体、同位素等多种相似分子的分离和纯化中均取得了卓越成效，并在聚合物及复杂生物样品的分析中展现出了巨大潜力。然而，尽管当前色谱固定相中使用的无序材料在性能、寿命和适用范围方面已达到很高水平，但关于OPM色谱柱在实际应用中的报道仍然有限。这主要受限于现有适用材料及加工工艺的制约，例如MOF、COF、POC等OPM固定相的成本较高，而3D打印等色谱柱制备技术的加工精度仍有待提升^［19，118］^，这些因素共同导致了难以制备出兼具长程有序、高分辨、低成本等特点的有序色谱固定相。此外，这些材料的颗粒尺寸、形貌和表面性质未能高度匹配色谱的要求，也阻碍了其大规模应用。近年来，国内外已出现一些新兴的OPM色谱柱，其中部分已成功实现商业化^［119］^。面对未来科学研究和工业生产中对分离精度日益增长的需求，OPM在色谱领域的应用仍面临诸多挑战，具体包括以下几个方面。

（1）分离性能的提升。更高的分辨率和分离度是色谱分离永恒的追求目标。当前OPM作为一种理想的分离材料，其对于某些特殊物质的分离仍受到限制。例如，OPM固定相难以应用于分离尺寸、形状和热力学性质几乎相同的分子；同时，对于具有复杂空间结构和多重相互作用的生物大分子，它们在色谱分离过程中的非特异性吸附问题也难以规避。目前，同位素气体的分离主要依赖于动力学量子筛分（KQS）和化学亲和量子筛分（CAQS）效应，尽管氢同位素分离技术已取得一定进展，但^3^He和^4^He、^16^O_2_和^18^O_2_、^12^CO_2_和^14^CO_2_等气体的分离仍是一大挑战。此外，含有蛋白质、核酸等大分子的复杂生物样品检测与分离也是色谱领域的难点，如何减少由亲/疏水等相互作用导致的非特异性吸附是固定相材料设计与合成中需要考虑的问题。因此，通过化学修饰引入特定的官能团、蛋白受体或互补核酸链，可以增强生物分子的分离选择性；同时，开发具有化学和拓扑各向异性、智能或响应性的多孔材料，以提高生物分子的分离度，将会是下一代生物分离OPM色谱的设计策略之一。

（2）复合材料的制备。不同种类的材料在性能上具有各自的优势，例如无机材料以其刚性见长，而有机材料则展现出良好的耐酸碱性。一般认为，由于多孔材料的合成工艺限制，难以同时实现高比表面积和孔径形貌的精确调控。因此，采用化学偶联或掺杂共混等手段将两种或多种材料相结合，以开发出性能更为优越的复合材料，已成为一种常见的设计策略。近年来，诸如MOF@SiO_2_、COF@Al_2_O_3_、COF@MOF等多种复合OPM的开发工作已有报道。目前，非化学键结合的BCP组装材料面临着机械强度不足、抗溶剂性能差等问题，亟待解决。将这类材料与无机粒子或网状交联材料进行复合，被视为改善其缺陷的有效途径。

（3）新兴技术的运用。新技术的出现有助于解决一些传统材料合成过程中存在的问题。近年来，多种新兴加工技术（如硅微加工和3D打印等^［118］^）被运用于OPM固定相的制备工艺中。特别是3D打印技术（又称增材制造），它借助计算机辅助设计（CAD）模型来构建所需的三维结构，被视为一种实现完美有序结构整体柱制备的可行方法^［120，121］^。凭借灵活的逐层构建工艺，3D打印技术能够突破传统色谱固定相设计的局限，创造出极为复杂的几何形状和拓扑结构，进而生产出专为分离科学中特定应用而设计的色谱柱。此外，3D打印技术还为阵列式柱色谱和微芯片色谱的制造提供了更为高效和便捷的构建途径。然而，3D打印技术目前仍面临可打印基材缺乏以及打印分辨率不足等挑战，有待后续深入研究与改进。此外，尽管双光子聚合（TPP）技术拥有高达0.1~1 µm的打印精度，但其打印速度较慢，若要制造全尺寸的色谱柱，将需要超长时间的打印过程。

（4）工业规模生产。现阶段，绝大多数有序多孔固定相的合成仍处于实验室研究阶段，且部分材料需在色谱柱等小型装置内部原位生成，导致生产规模受限。面对工业及市场中日益增长的分离应用需求，如何实现色谱OPM的低成本、高效率、大批量工业规模生产是目前亟待解决的问题。在工业生产中，确保不同批次产品间的稳定性至关重要，因此实现不同批次产品的可重复性是大规模生产时必须考虑的重要因素。此外，在多台微流控装置的并行化中，强耦合现象使得泡沫和乳液滴的放大生产变得困难，其界面稳定性成为多孔材料制备过程中的关键挑战^［122］^。此外，多设备和多流程的协同操作，以及大型设备中的反应热力学和动力学问题，也是化工设计过程中需要综合考虑的重要因素。
